# Discussion on Water Condensation in Membrane Pores during CO_2_ Absorption at High Temperature

**DOI:** 10.3390/membranes10120407

**Published:** 2020-12-09

**Authors:** Zhe Phak Chan, Lin Li, Guodong Kang, Norfaizah Ab Manan, Yiming Cao, Tonghua Wang

**Affiliations:** 1 School of Chemical Engineering, Dalian University of Technology, Dalian 116023, China; chanzhephak@petronas.com (Z.P.C.); lilin121@dlut.edu.cn (L.L.); 2 PETRONAS Research Sdn Bhd, Bangi 43000, Malaysia; norfaizah_amanan@petronas.com; 3 Dalian National Laboratory for Clean Energy, Dalian Institute of Chemical Physics, Chinese Academy of Science, Dalian 116023, China; ymcao@dicp.ac.cn

**Keywords:** capillary condensation, membrane contactor, CO_2_ absorption, membrane wetting, high temperature

## Abstract

Water condensation is a possible cause of membrane wetting in the operation of membrane contactors, especially under high-temperature conditions. In this study, water condensation in pores of polytetrafluoroethylene (PTFE) hollow fiber membranes was investigated during high-pressure CO_2_ absorption around 70 °C. It was found that the liquid accumulation rate in the treated gas knock-out drum was constant during continuous operation for 24 h when all experimental conditions were fixed, indicating a stable degree of membrane wetting. However, as the operating parameters were changed, the equilibrium vapor pressure of water within membrane pores could change, which may result in a condensation-conducive environment. Water condensation in membrane pores was detected and proven indirectly through the increase in liquid accumulation rate in the treated gas knock-out drum. The Hagen–Poiseuille equation was used to correlate the liquid accumulation rate with the degree of membrane wetting. The degree of membrane wetting increased significantly from 1.8 × 10^−15^ m^3^ to 3.9 × 10^−15^ m^3^ when the feed gas flow rate was reduced from 1.45 kg/h to 0.40 kg/h in this study due to water condensation in membrane pores. The results of this study provide insights into potential operational limitations of membrane contactor for CO_2_ absorption under high-temperature conditions.

## 1. Introduction

CO_2_ removal from gas streams is a common operation in the chemical industry. Many methods, including low-temperature distillation (cryogenic separation), membrane separation, adsorption, and absorption, have been used in CO_2_ removal. Membrane contactors, which combine the conventional absorption process with membrane technology, is considered one of the promising means for natural gas purification [1] and flue gas carbon capture [2,3]. The advantages offered by this technology include a larger specific contact area, higher mass transfer coefficient, a smaller size, and less operational problems such as foaming, channeling, and flooding [4]. Various studies have been conducted on membrane materials [5], absorbent types [6], operating conditions [7], and process modeling [8].

Membrane wetting, which can lead to partially or fully liquid-filled membrane pores, a condition where CO_2_ has to diffuse through a liquid phase instead of a gas phase, is a major challenge for this technology. This phenomenon will increase mass transfer resistance across the membrane, which is often observed as CO_2_ flux reduction in experiments [9,10]. Due to this reason, the study of membrane wetting and its mechanism is of great significance for the application of membrane contactors in CO_2_ absorption.

Generally, membrane wetting can happen via two mechanisms, which are direct liquid penetration into membrane pores due to the pressure difference between the gas phase and the liquid phase, and spontaneous liquid infiltration into membrane pores over time for materials with relatively low hydrophobicity (contact angle less than 90°). The first mechanism is usually described by the Young–Laplace equation [11], where liquid entry pressure (LEP) is defined as the liquid-gas pressure difference required for liquid to penetrate into membrane pores. As shown by Equation (1) below, LEP is dependent on three important parameters, which include surface tension of solvent (σ), the contact angle between solvent and membrane (θ), and the maximum pore radius ($r_{max}$).

(1) $$LEP=\;-\frac{2\sigma cos\theta }{r_{max}}$$

This equation explains the reason why membrane wetting in liquid absorbent with lower surface tension, such as with amine solutions, is more severe than water [12], and membranes with smaller pore sizes show better resistance to wetting. LEP can be lowered down due to membrane pores enlargement, as shown by Fang et al. in a study using polypropylene (PP) membranes over 480 h [13]. Similar morphology changes on low-density polypropylene (LDPE) membranes were also observed by Mosadegh–Sedghi et al. when it was in contact with amines [14]. These observations are good examples of membrane wetting via a second mechanism. PTFE hollow fiber membranes exhibited intrinsic hydrophobicity and excellent chemical stability, and therefore are less likely to have increased membrane wetting over time due to this reason [15].

Besides the two mechanisms described above, there might be a third mechanism for membrane wetting to happen. Jo et al. [16] described a mechanism on how condensation could happen on superhydrophobic surfaces, which eventually led to the disappearance of its superhydrophobicity. They pointed out that for a superhydrophobic surface with micro/nanostructures, there was a critical gap size (gap between micro/nanostructures) where condensation could happen in the gap under a certain vapor saturation level. The relationship between vapor saturation level and critical gap size is described by Kelvin’s equation

(2) $$2\sigma cos\theta =\;-RT/v_{l}\ln\left(\frac{P_{v}}{P_{sat}}\right)\cdot x_{crit}$$

where $x_{crit}$ is the gap size between nanostructures, σ is surface tension, θ is intrinsic contact angle, R is gas constant, $v_{l}$ is liquid molar volume, $P_{v}$ is vapor pressure, and $P_{sat}$ is saturated vapor pressure. When Kelvin’s equation is applied to porous membranes, $x_{crit}$ is equivalent to the average diameter of membrane pores [11], $P_{v}$ refers to the equilibrium vapor pressure of water within membrane pores, and θ refers to contact angle within membrane pores (intrinsic contact angle). For membranes with <90° intrinsic contact angle, $P_{v}$ is lower than $P_{sat}$; therefore, capillary condensation tends to happen in membrane pores. For membranes with >90° intrinsic contact angle, $P_{v}$ is higher than $P_{sat}$; therefore, condensation tends not to happen inside membrane pores under normal conditions.

At present, most membrane contactor studies are conducted at low temperatures with negligible water content in the feed gas, in which water vapor pressure is too low for water condensation to occur to a noticeable extent. Recently, Villeneuve et al. investigated the impact of water vapor condensation on the performance of hollow fiber membrane contactors in CO_2_ absorption using a monoethanolamine (MEA) solution [11]. The feed gas was humidified before entering into the membrane contactor. They concluded that water vapor condensation occurs in the gas phase or at the gas-membrane surface inside the fiber lumen, but not in the membrane pores. In other words, membrane wetting due to condensation may not happen. However, this is a conclusion obtained under stable experimental conditions at a relatively low temperature (40 °C). When there is a parameter change that leads to $P_{v}$ reduction during an experiment, there is a possibility that water vapor in membrane pores does not have sufficient time to diffuse out (the heat transfer is rapid while the mass transfer is slow [11]), and consequently, condensation may happen in membrane pores due to oversaturation, regardless of its intrinsic contact angle. This can be more severe when the temperature is high, and water vapor is abundant. High absorption temperature (70–75 °C) is required in a semi-lean CO_2_ absorption column in order to minimize the amount of energy required in heating up rich amine for regeneration. Therefore, it is necessary to explore the water condensation in CO_2_ absorption process using membrane contactors at relatively high temperatures.

The aim of this study is to evaluate whether condensation can occur in PTFE membrane pores during high-temperature CO_2_ absorption experiments. Experimental parameters, including feed gas flow rate, liquid flow rate, and liquid temperature, were changed in the middle of experiments to simulate the operational disturbance in a natural gas processing plant. Equilibrium vapor pressures of water in membrane pores were calculated using Kelvin’s equation to evaluate whether a condensation-conducive environment could exist when operational changes were introduced. Condensation in membrane pores was detected indirectly through monitoring of liquid accumulation rate in the treated gas knock-out drum. The Hagen–Poiseuille equation was used to correlate the liquid accumulation rate with the degree of membrane wetting. The outcome of this study provides insights into the potential operational limitations of membrane contactors for high-temperature CO_2_ absorption.

## 2. Materials and Methods

### 2.1. Materials

Pre-mixed CO_2_-N_2_ gas cylinders at the required composition were used instead of natural gas in this study, and the gas compositions of feed gas and treated gas were determined using gas chromatography (Agilent 490 Micro GC, Petaling Jaya, Malaysia). An aqueous solution of amine (36 wt%) were prepared with methyldiethanolamine (MDEA) and piperazine (PZ), which were purchased from Sigma Aldrich (Subang, Malaysia). The membrane module was manufactured in the Dalian Institute of Chemical Physics (China), where PTFE hollow fibers were used. The detailed information on PTFE hollow fibers and membrane module is listed in Table 1.

### 2.2. CO_2_ Absorption Experiments

**Figure 1:**
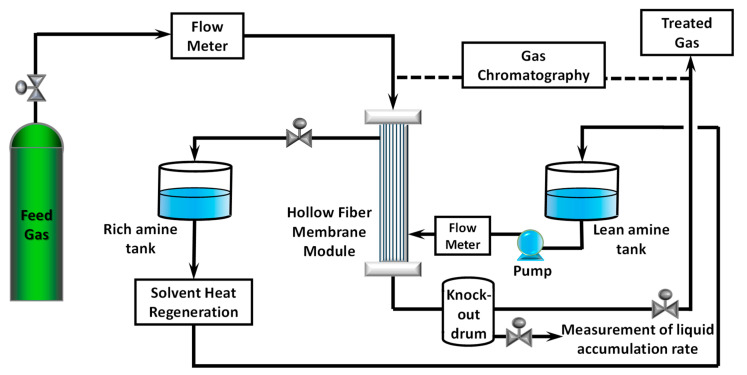
Schematic diagram of the membrane contactor test rig.

CO_2_ removal experiments were conducted in a high-pressure test rig. It was equipped with a programmable logic controller, online GC, automated pressure control valves, safety relief valves, temperature transmitters, pressure transmitters, flow transmitters, and level transmitters. The schematic diagram of the test rig is shown in Figure 1. Rich amine is continuously heat-regenerated and sent to a semi-lean amine tank.

CO_2_−N_2_ mixed gas was passed to the tube side of the hollow fiber membrane at 0.40–1.45 kg/h, while lean absorbent was pumped to the shell side at 8.00–12.20 kg/h. Gas flow and liquid flow were counter currents in the membrane module. The water vapor content of the mixed gas was measured using a dew point meter prior to the experiment. The flow rate of mixed gas was controlled using an electrical automatic control valve (PID (proportional integral derivative) tuned to give ≤± 0.015 MPa fluctuation), while the flow rate of lean absorbent was controlled by adjusting the pump stroke of a diaphragm meter pump equipped with a dampener. CO_2_ content in feed gas was 26% to reflect the feed gas of a natural gas processing plant in Malaysia. The temperature of mixed gas was maintained at room temperature. The pressure of the system was increased to about 5.3 MPa using control valves at both liquid outlet and gas outlet. Liquid pressure was kept slightly higher to avoid mixed gas from bubbling into the shell side. Knock-out drum after the membrane module was used to collect the liquid that exited membrane module together with treated gas. Each experiment was conducted for at least 30 min to achieve a stable condition. Pressure, temperature, and flow rates were logged automatically in 30 s intervals. Initial experimental conditions are listed in Table 2.

## 3. Results and Discussion

### 3.1. High-Temperature CO_2_ Removal Experiment

**Figure 2:**
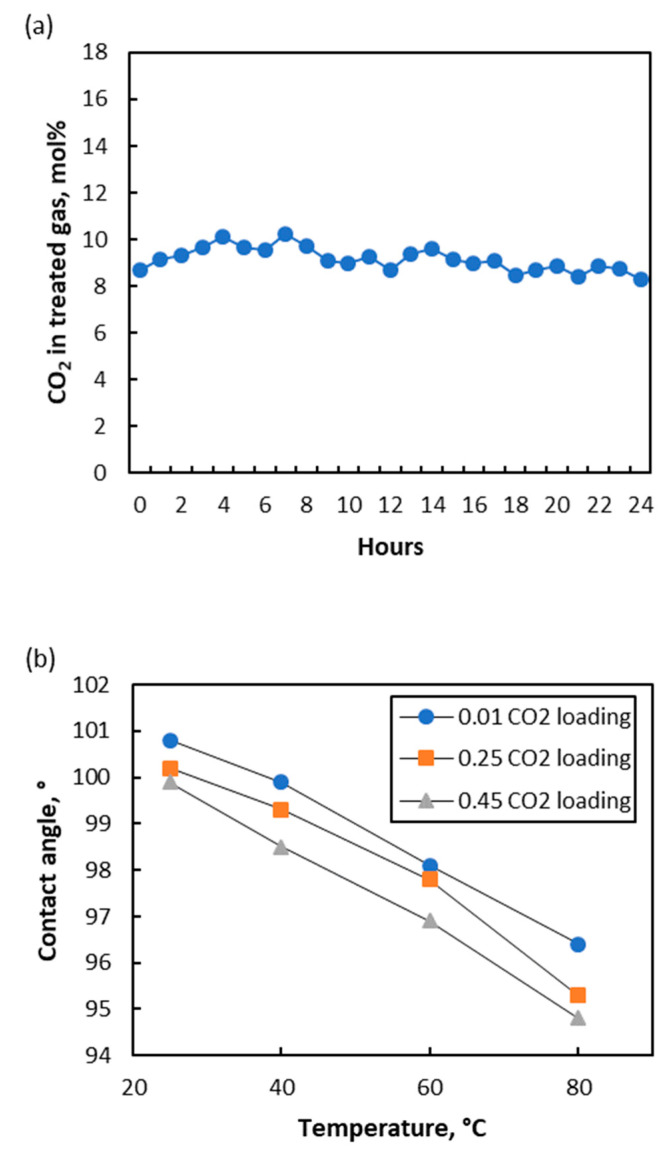

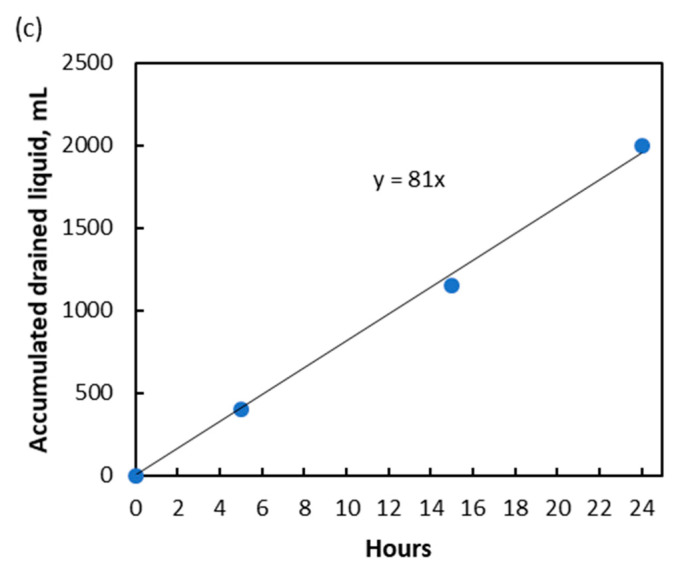
Continuous experiments with 26.0% CO_2_ content in inlet gas. (**a**) Trend of treated gas CO_2_ content; (**b**) Contact angle between solvent and membrane at various temperatures; (**c**) Accumulated drained liquid in knock-out drum with time.

CO_2_ removal experiment at high temperature was conducted under the experimental conditions listed in Table 2. Figure 2a shows the trend of treated gas CO_2_ content for a period of 24 h. It can be seen that CO_2_ removal performance was stable under high pressure and high absorption temperature, showing no sign of performance deterioration. To the author’s best knowledge, this is the first study that reports high-temperature CO_2_ absorption using membrane contactor, while high-pressure CO_2_ absorption using membrane contactor has recently been reported [17,18,19]. Liquid accumulation in the treated gas knock-out drum was observed due to the wetting of several large membrane pores during the experiment. This was expected as the contact angle slightly reduced at higher absorption temperature, as shown in Figure 2b. For the amine solution with 0.45 mol/mol CO_2_ loading, the value of −cos*θ* reduced from 0.174 to 0.087 when the contact angle (*θ*) reduced from 100° at 25 °C to 95° at 80 °C. Based on Equation (1), several membrane pores larger than 0.23 µm (radius) were expected to be wetted, which constituted only a small part of total membrane pores as the average membrane pore size was 0.10 µm (radius). Moreover, lower solvent viscosity at higher absorption temperature also led to an increase in the rate of solvent penetration through the wetted membrane pores, making membrane pores wetting easier to be detected. It was reflected in the liquid accumulation in the treated gas knock-out drum, which was not detected when the experiment was conducted at room temperature.

The liquid accumulation in the knock-out drum was not due to water vapor condensation as the outlet gas only contained 554 ppmv of water vapor, which was still far from the saturation level. The accumulated liquid was drained periodically for quantification. It was found that the total volume of the drained liquid increased linearly with time, as shown in Figure 2c. The slope of the trend line indicated that the liquid accumulation happened at a constant rate of 81 mL/h, suggesting a constant number of liquid-penetrated membrane pores or a constant degree of membrane wetting during the experiment when the experimental parameters were fixed. Unlike the membrane distillation process, where penetrated membrane pores will increase salt breakthrough to permeate side and subsequently impacting quality of the distilled water [12], the presence of liquid in gas side of a gas-liquid membrane contactor is not a problem as it can be knocked-out easily.

The liquid accumulation rate was affected by a few parameters, as shown by the Hagen–Poiseuille equation [20] below.

(3) $$Q=\left(\frac{n\pi R^{4}}{8L}\right)\frac{\Delta P}{\mu }$$

ΔP refers to the trans-membrane pressure difference, μ is the dynamic viscosity of amine solution, L is the average length of liquid-penetrated pores, Q is the liquid accumulation rate, R is the average radius of liquid-penetrated pores, and n is the number of liquid-penetrated pores. Based on this equation, changes in experimental parameters that affected ΔP and μ could change liquid penetration rate. $n\pi R^{4}/8L$ is constant as long as a number of liquid–penetrated pores remain constant; therefore, this value could be used to indicate the degree of membrane wetting. The value of $n\pi R^{4}/8L$ should be zero when there is no membrane wetting. Table 3 below shows calculation parameters and degree of membrane wetting for the 24 h continuous experiment conducted. ΔP at the liquid inlet and liquid outlet were different due to different pressure drop in gas flow and liquid flow.

### 3.2. Changes in Feed Gas Flow Rate

As mentioned above, when there is a parameter change that leads to $P_{v}$ reduction during an experiment, there is a possibility that water vapor in membrane pores does not have sufficient time to diffuse out, and water condensation may happen in membrane pores due to oversaturation. Therefore, the operation parameters, including the feed gas flow rate, the liquid flow rate, and liquid temperature, were adjusted in the middle of an experiment to change the equilibrium status of water vapors in membrane pores. The resulting indirect water condensation observation was analyzed and discussed.

**Figure 3:**
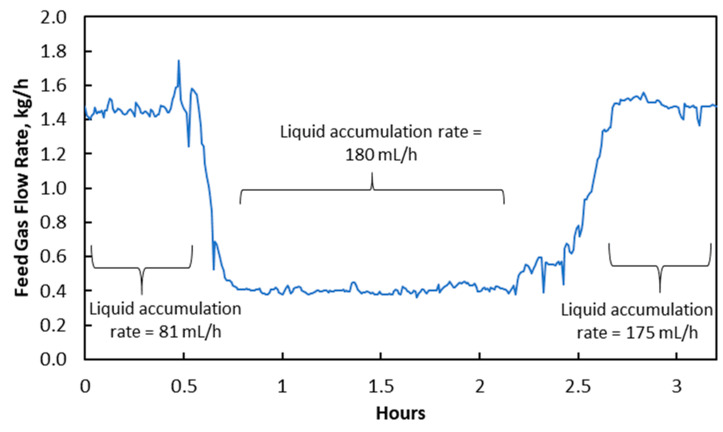
Changes in feed gas flow rate and its impact on liquid accumulation rate in treated gas knock-out drum.

The feed gas flow rate was rapidly reduced from 1.45 kg/h to 0.40 kg/h in an experiment, which resulted in an increase of liquid accumulation rate from 81 mL/h to 180 mL/h shown in Figure 3. This was mainly due to an increase in membrane wetting from 1.8 × 10^−15^ m^3^ to 3.9 × 10^−15^ m^3^, as indicated in Table 4. A slight increase in log mean ΔP was caused by a slight reduction in gas phase pressure drop. It had a relatively minor role in causing the liquid accumulation rate to increase and was less likely the main reason. Instead, the increase in liquid accumulation of membrane wetting should be due to the water condensation in membrane pores.

**Figure 4:**
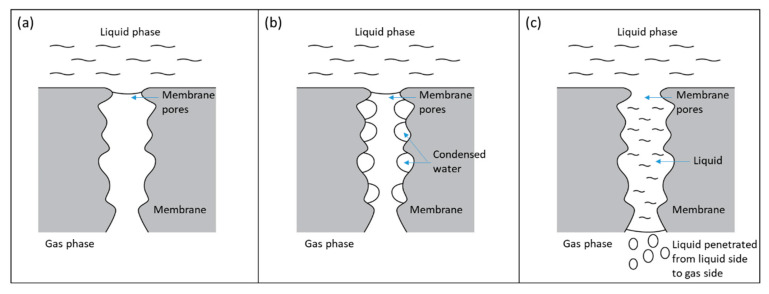
Membrane wetting mechanism caused by water condensation. (**a**) Dry membrane pores. (**b**) Membrane pores with condensed water droplets. (**c**) Membrane pores fully filled with condensed water and formed a liquid bridge for a liquid to go into the gas side of the hollow fiber membranes.

When feed gas flow rate was reduced, the heat released from exothermic reaction reduced and resulted in lower membrane pore temperature and equilibrium water vapor pressure ($P_{v}$), which was calculated using Equation (2). Table 5 shows that $P_{v}$ reduced significantly from 60.36 kPa to 46.12 kPa. However, the actual water vapor pressure in membrane pores could not rapidly be reduced because it was limited by the diffusion rate. The water vapor diffusion rate in membrane pores was 3.18 × 10^−2^ mol/(m^2^·s), estimated from Fuller–Schettler–Giddings correlation and effective diffusion coefficient [21,22,23]. The condition where the actual water vapor pressure in membrane pores exceeded the $P_{v}$, created a high potential for condensation to occur inside membrane pores. This condensation-conducive environment caused some of the membrane pores to be fully filled by condensed water, allowing increased liquid penetration from the shell side to the tube side of hollow fiber membranes, as illustrated in Figure 4. Jo et al. [16] conducted a study in which they cooled a superhydrophobic PTFE surface in an ESEM (environment scanning electron microscope) with constant vapor pressure and observed water condensation from inside the hydrophobic interstices. Kato et al. [24] carried out a study on water condensation in the hydrophobic microporous layer (MPL) of polymer electrolyte fuel cells and showed that water condensation and accumulation could happen in a MPL. Guillen-Burrieza et al. [12] also reported similar phenomena in a membrane distillation study, in which temperature reduction during an experiment led to rapid conductivity increase, which was a convenient indication of membrane wetting in a membrane distillation system.

Then we adjusted the feed gas flow rate back to 1.45 kg/h. However, it was found that the liquid accumulation rate did not return to the original level. Instead, it only reduced slightly from 180 mL/h to 175 mL/h, which was due to the ΔP reduction. This phenomenon suggests that membrane wetting caused by water condensation in membrane pores is not reversible by simply returning to the original operating conditions. The membrane pores will remain liquid-penetrated until the membrane is washed and dried. Therefore, in order to minimize the impact of feed gas flow rate reduction, the changes of operation parameters should be carried out gradually to allow more time for excessive water vapor in membrane pores to diffuse out. In fact, when the adjustment of the feed gas flow rate was very slow (for about 2 h), the increase of liquid accumulation rate was not observed. It should also be noted that if physical solvent was used, gas flow reduction might not cause membrane wetting, as the change in temperature coming from increased or reduced exothermic reaction would be minimal [25]. In addition, a higher contact angle between liquid droplet and membrane would also be helpful to reduce membrane wetting by having a slower growth rate of condensed water droplets, as shown in an experiment carried out by Leach et al. [26]. It was due to the reason that the nucleation barrier in heterogeneous water condensation increased with the contact angle, based on classical nucleation theory.

### 3.3. Changes in Liquid Flow Rate

**Figure 5:**
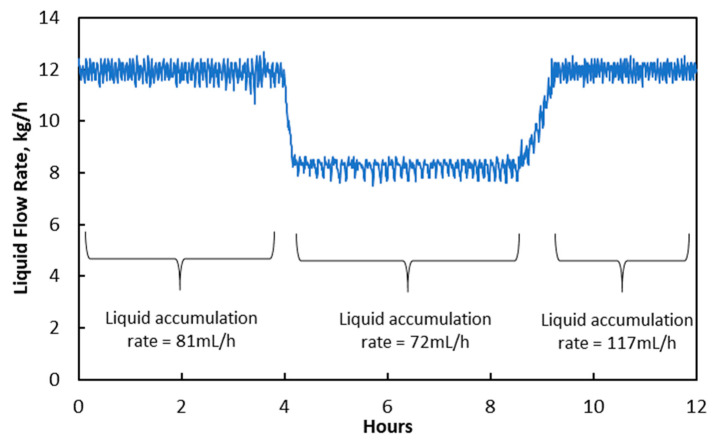
Changes in liquid flow rate and its impact on liquid accumulation rate at treated gas knock-out drum.

Then, the effect of changes in liquid flow rate on liquid accumulation rate was investigated. The liquid flow rate was adjusted from 12.2 kg/h to 8.0 kg/h, and the experimental results are shown in Figure 5. It can be seen that the liquid accumulation rate reduced slightly from 81 mL/min to 72 mL/min due to the decreased ΔP as shown in Table 6. The average temperature at liquid inlet increased from 73 °C to 77 °C due to an intensified exothermic reaction, causing the increase in $P_{v}$ as shown in Table 7. The increase in $P_{v}$ could evaporate condensed water in membrane pores but could not reduce the number of “liquid-penetrated” pores, as liquid flow in those membrane pores was continuous.

However, when the liquid flow rate was increased back to 12.2 kg/h at the eighth hour, the liquid accumulation rate increased to 133 mL/h, indicating a higher degree of membrane wetting (2.6 × 10^−15^ m^3^) compared to the original condition (1.8 × 10^−15^ m^3^). Temperature and $P_{v}$ reduced when the liquid flow rate was increased. This created a conducive condition for water condensation to occur inside membrane pores. Rongwong et al. [27] reported that membrane wetting could increase with liquid velocity due to an increase in ΔP. This is less likely in this study as the ΔP merely increased back to the original level when the liquid flow rate was increased back to 12.2 kg/h, and the magnitude of ΔP increase was only 1000 Pa.

### 3.4. Reduction of Absorption Temperature

**Figure 6:**
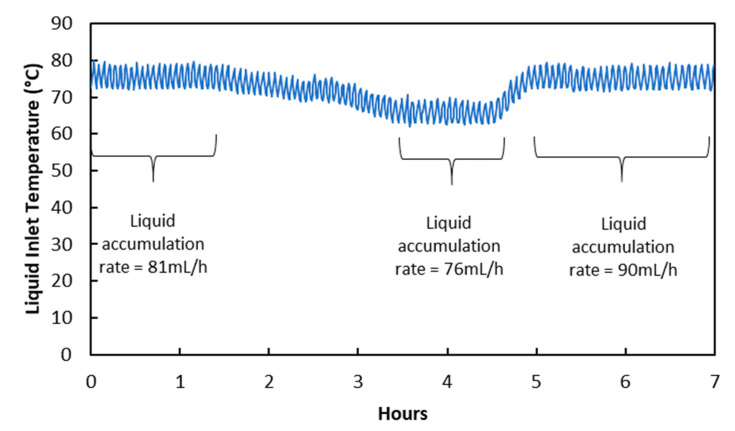
Changes in liquid temperature and its impact on liquid accumulation rate in treated gas knock-out drum.

Results and discussions in Section 3.3 and Section 3.4 show that water condensation in membrane pores happened due to temperature change when flow rates were changed. This suggests that condensation in membrane pores can also happen when the temperature in the membrane module is changed directly. In fact, this observation was reported in a membrane distillation study conducted by Rezaei et al. [28]. Figure 6 below shows the effect of temperature reduction on the liquid accumulation rate in the treated gas knock-out drum. Liquid accumulation rate reduced from 81 mL/h to 76 mL/h when liquid inlet temperature was reduced from 75 °C to 66 °C by reducing the temperature of the water bath heat exchanger. The reduction in liquid accumulation rate was due to liquid viscosity increase rather than membrane wetting decrease, as shown in Table 8. In fact, membrane wetting increased slightly due to water condensation in membrane pores as a result of $P_{v}$ reduction, as shown in Table 9. It will be difficult to arrive at this conclusion without an analysis using the Hagen–Poiseuille equation. When the temperature was adjusted back to the original level, liquid viscosity returned to the original value as well. This allowed the liquid accumulation rate to reflect the slight increase in membrane wetting.

Temperature reduction was slower compared to flow rates reduction in Section 3.3 and Section 3.4, and the increase in membrane wetting was also smaller in magnitude. This shows that gradual change in temperature or parameters affecting temperature could reduce water vapor condensation in membrane pores. There was more time for water vapor in membrane pores to diffuse out when the temperature was reduced, resulting in only mild supersaturation. Besides that, the condensed water droplet growth rate was slow due to the low effective temperature difference between water vapor and condensation surface [29]. This finding provides a good basis for other operational considerations such as start-up, shut-down, turn-down, and maintenance of membrane contactors.

## 4. Conclusions

Performance stability of PTFE membrane contactor for high-temperature CO_2_ absorption was proven for 24 h. Liquid penetration or membrane wetting happened to a small number of membrane pores during the experiments, and the number of liquid-penetrated membrane pores was constant throughout the experiment, indicated by constant liquid accumulation rate in the treated gas knock-out drum. Calculation showed that equilibrium vapor pressure of water in membrane pores ($P_{v}$) fluctuated together with flow rates and temperature, which created either a condensation-conducive or evaporation-conducive environment. A condensation-conducive environment was created in membrane pores when there was direct or indirect temperature reduction, which led to excessive water condensation. Membrane pores that had been fully filled by condensed water allowed liquid penetration from the liquid side into the gas side, and the degree of membrane wetting was evaluated using the Hagen–Poiseuille equation. Results from this study offer a better understanding of membrane wetting due to water condensation in membrane pores. It also provides important insights into potential operational limitations of membrane contactors for semi lean CO_2_ absorption, as well as its potential solutions.

## Figures and Tables

**Table 1:** Details of polytetrafluoroethylene (PTFE) hollow fibers and membrane module.

Parameter	Value
Fiber outer diameter (mm)	0.93
Fiber inner diameter(mm)	0.42
Porosity of fibers (%)	40
Average pore radius (μm)	0.10
Maximum pore radius (μm)	0.38
Number of hollow fibers in membrane module	120
Module inner diameter (mm)	15
Packing density (%)	46
Effective length of membrane module (m)	1
Membrane area (m^2^)	0.35

**Table 2:** Initial experimental conditions.

Parameters	Value
Inlet gas CO_2_ content, %	26
Inlet gas pressure, MPa	5.310 ± 0.015
Inlet gas temperature, K	301.0 ± 0.1
Inlet gas flow rate, kg/h	1.45 ± 0.05
Inlet gas water vapor content, ppmv	28 ± 1
Outlet gas pressure, MPa	5.298 ± 0.015
Inlet liquid CO_2_ loading, mol CO_2_/mol amine	0.40–0.42
Inlet liquid pressure, MPa	5.334 ± 0.015
Inlet liquid temperature, K	346 ± 3.5
Inlet liquid flow rate, kg/h	12.20 ± 0.60
Inlet liquid amine concentration, wt%	36

**Table 3:** Calculation parameters for the degree of membrane wetting ($n\pi R^{4}/8L$).

Parameters	24 h Experiment
Q, m^3^/s	2.3 × 10^−8^
ΔP at liquid inlet, Pa	3.5 × 10^4^
ΔP at liquid outlet, Pa	2.0 × 10^4^
Log mean ΔP, Pa	2.7 × 10^4^
μ at liquid inlet, Pa·s	2.3 × 10^−3^
μ at liquid outlet, Pa·s	2.1 × 10^−3^
Average μ, Pa·s	2.2 × 10^−3^
ΔP/μ, s^−1^	1.2 × 10^7^
$n\pi R^{4}/8L$, m^3^	1.8 × 10^−15^

**Table 4:** Changes in the degree of membrane wetting when feed gas flow rate was reduced.

Parameters	Original Gas Flow 1.45 kg/h	Reduced Gas Flow 0.40 kg/h	Return to Original Gas Flow 1.45 kg/h
Q, m^3^/s	2.3 × 10^−8^	5.0 × 10^−8^	4.9 × 10^−8^
Log mean ΔP, Pa	2.7 × 10^4^	2.9 × 10^4^	2.7 × 10^4^
Average μ, Pa·s	2.2 × 10^−3^	2.2 × 10^−3^	2.2 × 10^−3^
$n\pi R^{4}/8L$, m^3^	1.8 × 10^−15^	3.9 × 10^−15^	3.9 × 10^−15^

**Table 5:** Changes in water vapor pressures when feed gas flow rate was reduced.

Parameters	Feed Gas Flow 1.45 kg/h	Feed Gas Flow 0.40 kg/h
Liquid inlet temperature, K	346.4	346.4
Liquid outlet temperature, K	355.8	349.5
Liquid inlet H_2_O $P_{sat}$, kPa	32.43	32.43
Liquid inlet H_2_O $P_{v}$, kPa	32.45	32.45
Liquid outlet H_2_O $P_{sat}$, kPa	60.33	46.10
Liquid outlet H_2_O $P_{v}$, kPa	60.36	46.12

**Table 6:** Changes in the degree of membrane wetting when the liquid flow rate was adjusted.

Parameters	Original Liquid Flow 12.2 kg/h	Reduced Liquid Flow 8.0 kg/h	Return to Original Liquid Flow 12.2 kg/h
Q, m^3^/s	2.3 × 10^−8^	2.0 × 10^−8^	3.3 × 10^−8^
Log mean ΔP, Pa	2.7 × 10^4^	2.6 × 10^4^	2.7 × 10^4^
Average μ, Pa·s	2.2 × 10^−3^	2.0 × 10^−3^	2.2 × 10^−3^
$n\pi R^{4}/8L$, m^3^	1.8 × 10^−15^	1.6 × 10^−15^	2.6 × 10^−15^

**Table 7:** Changes in water vapor pressures when the liquid flow rate was reduced.

Parameters	Liquid Flow 12.20 kg/h	Liquid Flow 8.00 kg/h
Liquid inlet temperature, K	346.4	350.2
Liquid outlet temperature, K	355.8	360.0
Liquid inlet H_2_O $P_{sat}$, kPa	32.43	38.08
Liquid inlet H_2_O $P_{v}$, kPa	32.45	38.11
Liquid outlet H_2_O $P_{sat}$, kPa	60.33	72.21
Liquid outlet H_2_O $P_{v}$, kPa	60.36	72.25

**Table 8:** Changes in the degree of membrane wetting when the temperature was adjusted.

Parameters	Original Temperature 75 °C	Original Temperature 66 °C	Return to Original Temperature 66 °C
Q, m^3^/s	2.3 × 10^−8^	2.1 × 10^−8^	2.5 × 10^−8^
Log mean ΔP, Pa	2.7 × 10^4^	2.7 × 10^4^	2.7 × 10^4^
Average μ, Pa·s	2.2 × 10^−3^	2.4 × 10^−3^	2.2 × 10^−3^
$n\pi R^{4}/8L$, m^3^	1.8 × 10^−15^	1.9 × 10^−15^	2.0 × 10^−15^

**Table 9:** Changes in water vapor pressures with liquid inlet temperature.

Parameters	Liquid Inlet 75 °C	Liquid Inlet 66 °C
Liquid inlet temperature, K	348.2	339.2
Liquid outlet temperature, K	357.2	349.8
Liquid inlet H_2_O $P_{sat}$, kPa	35.03	23.66
Liquid inlet H_2_O $P_{v}$, kPa	35.05	23.67
Liquid outlet H_2_O $P_{sat}$, kPa	63.96	47.56
Liquid outlet H_2_O $P_{v}$, kPa	63.99	47.58
